# Subspecies in the Sarus Crane *Antigone antigone* revisited; with particular reference to the Australian population

**DOI:** 10.1371/journal.pone.0230150

**Published:** 2020-04-16

**Authors:** Timothy D. Nevard, Martin Haase, George Archibald, Ian Leiper, Robert N. Van Zalinge, Nuchjaree Purchkoon, Boripat Siriaroonrat, Tin Nwe Latt, Michael Wink, Stephen T. Garnett

**Affiliations:** 1 Research Institute for the Environment and Livelihoods, Charles Darwin University, Darwin, Northern Territory, Australia; 2 Atherton Tablelands Foundation, Ravenshoe, Queensland, Australia; 3 AG Vogelwarte, Zoologisches Institut und Museum, Universität Greifswald, Greifswald, Germany; 4 International Crane Foundation, Baraboo, Wisconsin, United States of America; 5 Zoological Park Organisation, Dusit Bangkok, Thailand; 6 Faculty of Environmentand Resource Studies, Mahidol University, Salaya, Phutthamonthon Nakhon Pathom, Thailand; 7 Institut für Pharmazie & Molekulare Biotechnologie (IPMB), Heidelberg, Germany; National Taiwan Normal University, TAIWAN

## Abstract

Subspecies are often less well-defined than species but have become one of the basic units for legal protection. Evidence for the erection or synonymy of subspecies therefore needs to be founded on the best science available. Here we show that there is clear genetic disjunction in the Sarus Crane *Antigone antigone*, where previously the variation had appeared to be clinal. Based on a total sample of 76 individuals, analysis of 10 microsatellite loci from 67 samples and 49 sequences from the mitochondrial control region, this research establishes that the Australian Sarus Crane *A*. *a*. *gillae* differs significantly from both *A*. *a*. *antigone* (South Asia) and *A*. *a*. *sharpii* (Myanmar and Indochina). A single sample from the extinct Philippine subspecies *A*. *a luzonica* clustered with *A*. *a*. *gillae*, hinting at the potential for a more recent separation between them than from *A*. *a*. *antigone* and *A*. *a*. *sharpii*, even though *A*. *a*. *sharpii* is closer geographically. The results demonstrate that failure to detect subspecies through initial genetic profiling does not mean discontinuities are absent and has significance for other cases where subspecies are dismissed based on partial genetic evidence. It could also be potentially important for sourcing birds for reintroduction to the Philippines.

## Introduction

Species are defined along a continuum from emphasising phenotypic distinctiveness through to reproductive incompatibility [1] with over 30 definitions currently in use [2]. Subspecies are even less well defined and this is uneven amongst taxa. Broadly, subspecies represent geographically defined populations that are potentially incipient species, diagnosable by at least one heritable trait but still reproductively compatible [3]. While there have also been attempts to define subspecies statistically [4,5], debate continues [6,7] and the expectation that genetic analysis would resolve ambiguities has not eventuated. For example, while cetacean biologists are content to define subspecies quantitatively on the basis of mitochondrial DNA control region sequence data alone [8], this approach has been rejected for birds [9]; not least because there is often discordance between mitochondrial and nuclear DNA [10].

This is not merely an academic debate and definitions matter. A failure to recognise subspecies can mean they might be lost before being recognised as warranting conservation attention [11]; on the other hand, over-splitting increases the probability of genetic problems among the necessarily smaller populations identified [12]. Subspecies are, with species, the common currency of threatened species conservation in most jurisdictions [13] with the erection or synonymy of subspecies having legal, financial and social consequences. For example, had the US Fish and Wildlife Service followed Zink *et al*. [14] and decided that the California Gnatcatcher (*Polioptila c*. *californica*) did not warrant subspecies status, 80,000 ha of its critical coastal sage scrub habitat would have been released to development [15]. In the event they decided otherwise, on the basis that the best available scientific information did not support synonymy [16].

Following extensive fieldwork [17,18,19] involving significant observational and genetic study of Australian Sarus Cranes *Antigone a*. *gillae* [20], we hypothesised that further investigation of phylogeographic variation in the full range of Sarus Crane *Antigone antigone* (Linnaeus 1758) subspecies had the potential to change both the taxonomic treatment of Australian Sarus Cranes and the value given to different populations.

**Fig 1:**
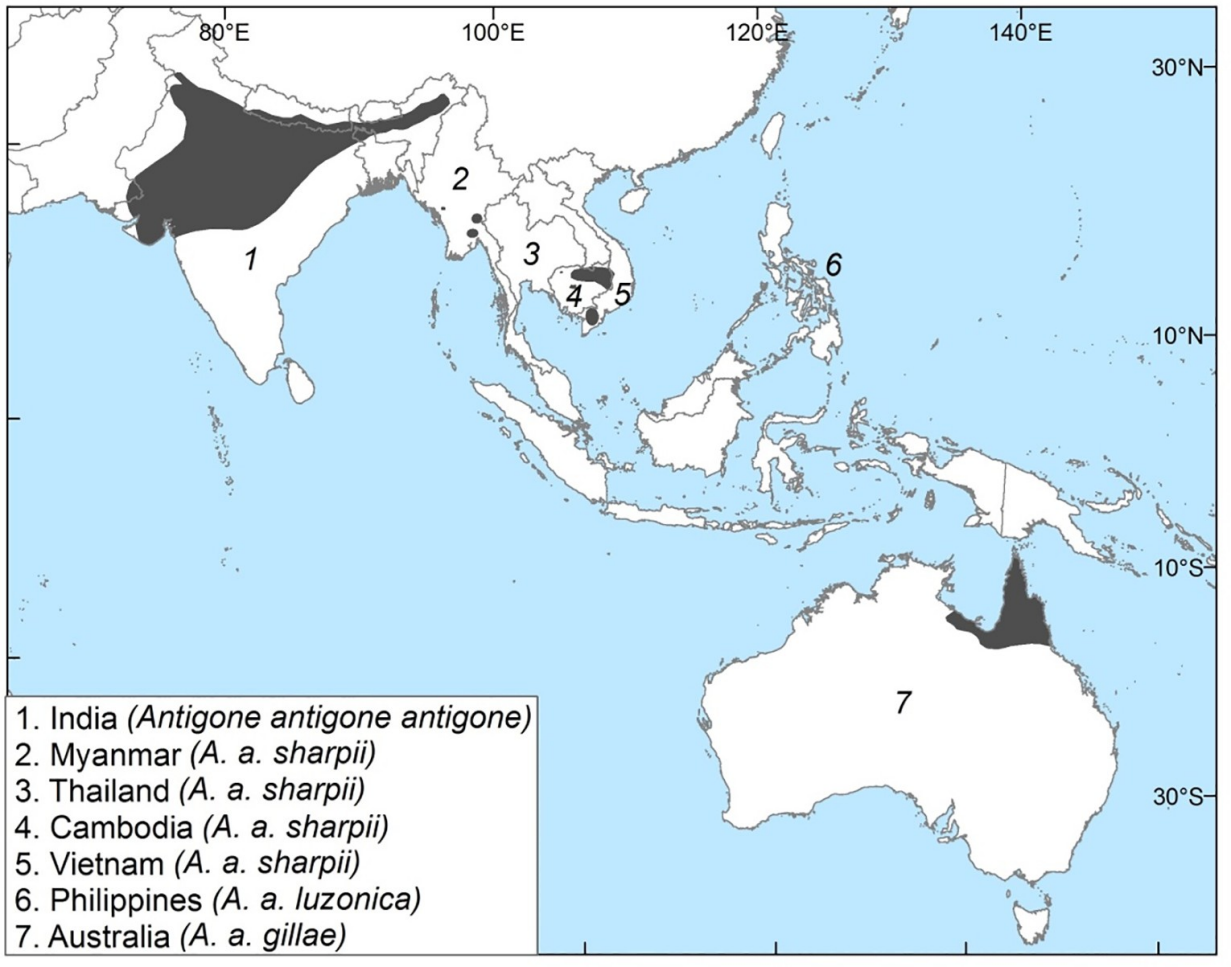
Global distribution of the Sarus Crane *A*. *antigone*, showing populations and subspecies [Distribution data derived from BirdLife International and NatureServe Bird Species Distribution Maps of the World [80], the Australian Bird Guide [81] and author contributions].

The Sarus Crane has geographically separate populations in southern Asia and Australia (Fig 1) that are believed to be geographically allopatric. As it is extinct in the Philippines and thought to be declining in some of its Asian range, particularly in Myanmar and Indochina [21,22], it is classed as Vulnerable by the IUCN [23]. Intraspecific variation within the species has been the subject of ongoing debate. Blyth and Tegetmeier [24] initially erected the Indian and Myanmar birds as distinct species, based on plumage (the Indian Sarus Crane has a white upper neck and tertials) and body size. Sharpe [25] retained this distinction but shortly afterwards Blanford [26] combined them into one species with two subspecies, *Grus antigone antigone* and *Grus antigone sharpii* respectively, a classification which has since endured. Hachisuka [27] described the (then extant) Philippine population as *Grus antigone luzonica*, sufficiently distinct from both *G*. *a*. *antigone* and *G*. *a*. *sharpii* to warrant subspecies status. Del Hoyo and Collar [28] dispute this and place the Philippine birds in *A*. *a*. *sharpii*. Sarus Cranes were observed in Australia in 1966, [29] and placed in *A*. *a*. *sharpii* but were subsequently described by Schodde [20] as a new subspecies *G*. *a*. *gillae*, on the basis of distinct plumage and a larger ear patch. Archibald (personal observation) noted that *A*. *a*. *gillae* also has different unison calls from both *A*. *a*. *antigone* and *A*. *a*. *sharpii*, helping to differentiate it from the sympatric Brolga *A*. *rubicunda*.

**Fig 2:**
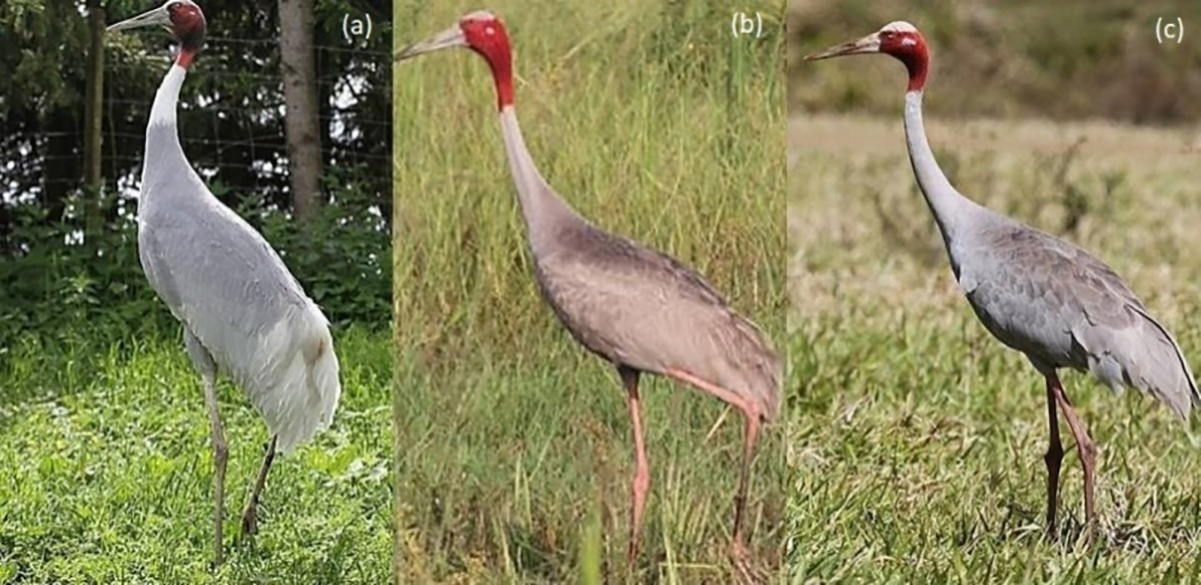
Extant Sarus Crane subspecies. (a) *antigone* South Asia (captive bird); (b) *sharpii* Myanmar and Indochina (wild Cambodian bird); (c) *gillae* Australia (wild Northeast Queensland bird). [Photographs T, Nevard (a) and (c); Robert van Zalinge (b)].

These subspecific arrangements, largely indicated by morphology (Fig 2), have not hitherto been strongly supported by genetic analyses. Application of molecular techniques to understand the subspecific arrangements of Sarus Cranes [30,31,32] suggested that colonisation of Australia by Sarus Cranes was relatively recent and there had been little differentiation of populations across their range [32].

Using neutral genetic information as a decisive basis for the recognition of morphologically defined subspecies has been rightly criticized [7]. Morphological variation and variation of standard genetic markers such as mitochondrial DNA or microsatellites do not have to correlate and lack of differentiation at these loci does not disprove taxonomic decisions based on other types of characters. Gavrilets [33] notes that despite gene flow, local selection may be sufficient to maintain differences. However, neutral genetic differentiation among populations that are also morphologically differentiated does indicate limited gene flow among these populations, supporting their taxonomic distinction. It is in this context that we have analysed the genetic differentiation of the morphologically defined subspecies of the Sarus crane, based on the largest sample size available so far.

Potential differentiation among populations is relevant for two reasons. First, as a contribution to the debate about using analyses of neutral genetic markers to synonymise or retain subspecies—particularly as to whether differentiated populations should continue to be treated as separate, valued and taxonomically recognised management units. Second, reintroduction of Sarus Cranes to the Philippines is being considered (J. C. Gonzales, personal communication), so an appropriate potential founding stock needs to be identified.

## Materials and methods

### Ethics approval

The research was carried out in accordance with Charles Darwin University Animal Ethics Committee’s approval A13019 and Queensland Department of Environment and Heritage Protection Scientific Purposes Permit WISP13984714. Where required, collection and carriage of samples to Greifswald University was undertaken under CITES permits: PWS2014-AU-001240, PWS2015-AU-000119, 000002/FD-2011 and KH1108.

### Samples

We secured sample material opportunistically from all four putative subspecies and most range countries (Tables 1& 2). Sample material varied from naturally shed and deliberately plucked feathers, to blood taken from live birds (both wild and captive) and tissue harvested from museum specimens (toe pad samples).

**Table 1:** Sources of Sarus Crane DNA used in analyses.

Subspecies	Country	Sample type	No. DNA samples	
			Nuclear	Mitochondrial
*antigone*	India	Blood	5	3
		Toe pad	3	1
*gillae*	Australia	Feather	25	20
*luzonica*	Philippines	Toe pad	1	-
*sharpii*	Cambodia	Brain	1	-
		Blood	12	6
		Oral swab	1	-
	Myanmar	Blood	11	11
	Thailand	Blood	8	8
Total			67	49

**Table 2:** Details of sample collections for Sarus Crane (Ssp: subspecies; M: microsatellites; S: sequences).

Code	Ssp	Country	Locality	Collection number	DNA source	M/S
Aus01	*gillae*	Australia	Gulf Plains	B1974	feather	M/S
Aus02	*gillae*	Australia	Gulf Plains	B1976	feather	M/--
Aus03	*gillae*	Australia	Gulf Plains	B1980	feather	M/S
Aus04	*gillae*	Australia	Gulf Plains	B1986	feather	M/--
Aus05	*gillae*	Australia	Gulf Plains	B1989	feather	M/--
Aus06	*gillae*	Australia	Gulf Plains	B2168	feather	M/--
Aus07	*gillae*	Australia	Gulf Plains	B2216	feather	M/S
Aus08	*gillae*	Australia	Gulf Plains	B2220	feather	M/--
Aus09	*gillae*	Australia	Gulf Plains	B2225	feather	M/--
Aus10	*gillae*	Australia	Gulf Plains	B2228	feather	M/--
Aus11	*gillae*	Australia	Gulf Plains	B2233	feather	M/--
Aus12	*gillae*	Australia	Gulf Plains	B2234	feather	M/--
Aus13	*gillae*	Australia	Gulf Plains	B2239	feather	M/--
Aus14	*gillae*	Australia	Gulf Plains	B2241	feather	M/S
Aus15	*gillae*	Australia	Gulf Plains	B2243	feather	M/--
Aus16	*gillae*	Australia	Gulf Plains	B2245	feather	M/--
Aus17	*gillae*	Australia	Gulf Plains	B2247	feather	M/--
Aus18	*gillae*	Australia	Gulf Plains	B2248	feather	M/--
Aus19	*gillae*	Australia	Gulf Plains	B2249	feather	M/--
Aus20	*gillae*	Australia	Gulf Plains	B2254	feather	M/S
Aus21	*gillae*	Australia	Gulf Plains	B2255	feather	M/--
Aus22	*gillae*	Australia	Gulf Plains	B2258	feather	M/S
Aus23	*gillae*	Australia	Gulf Plains	B2261	feather	M/--
Aus24	*gillae*	Australia	Gulf Plains	B2352	feather	M/--
Aus25	*gillae*	Australia	Gulf Plains	B2380	feather	M/--
Aus26	*gillae*	Australia	Gulf Plains	B1922	feather	--/S
Aus27	*gillae*	Australia	Gulf Plains	B1926	feather	--/S
Aus28	*gillae*	Australia	Gulf Plains	B1927	feather	--/S
Aus29	*gillae*	Australia	Gulf Plains	B1983	feather	--/S
Aus30	*gillae*	Australia	Gulf Plains	B2008	feather	--/S
Aus31	*gillae*	Australia	Gulf Plains	B2009	feather	--/S
Aus32	*gillae*	Australia	Gulf Plains	B2015	feather	--/S
Aus33	*gillae*	Australia	Gulf Plains	B2196	feather	--/S
Aus34	*gillae*	Australia	Gulf Plains	B2198	feather	--/S
Aus35	*gillae*	Australia	Gulf Plains	B2218	feather	--/S
Aus36	*gillae*	Australia	Gulf Plains	B2224	feather	--/S
Aus37	*gillae*	Australia	Gulf Plains	B2250	feather	--/S
Aus38	*gillae*	Australia	Gulf Plains	B2251	feather	--/S
Aus39	*gillae*	Australia	Gulf Plains	B2327	feather	--/S
Cam01	*sharpii*	Cambodia	Siem Reap, Angkor Centre for Conservation of Biodiversity	KHL15-ACCB-006-Br-RNA	brain	M/--
Cam02	*sharpii*	Cambodia	Tonle Sap floodplains	KHL16-ZALINGE-002	blood	M/--
Cam03	*sharpii*	Cambodia	Tonle Sap floodplains	KHL16-ZALINGE-003	blood	M/--
Cam04	*sharpii*	Cambodia	Mekong delta region	KHL16-ZALINGE-005	blood	M/--
Cam05	*sharpii*	Cambodia	Mekong delta region	KHL16-ZALINGE-006	blood	M/--
Cam06	*sharpii*	Cambodia	Tonle Sap floodplains	KHL16-ZALINGE-008	blood	M/S
Cam07	*sharpii*	Cambodia	Tonle Sap floodplains	KHL16-ZALINGE-009	blood	M/--
Cam08	*sharpii*	Cambodia	Tonle Sap floodplains	KHL16-ZALINGE-011	blood	M/S
Cam09	*sharpii*	Cambodia	Siem Reap, Angkor Centre for Conservation of Biodiversity	KHL16-ZALINGE-KH003	blood	M/S
Cam10	*sharpii*	Cambodia	Tonle Sap floodplains	KHL16-ZALINGE-R/W	blood	M/S
Cam11	*sharpii*	Cambodia	Tonle Sap floodplains	KHL16-ZALINGE-001	blood	M/S
Cam12	*sharpii*	Cambodia	Siem Reap, Angkor Centre for Conservation of Biodiversity	KHL16-ZALINGE-KH001	blood	M/--
Cam13	*sharpii*	Cambodia	Siem Reap, Angkor Centre for Conservation of Biodiversity	KHL16-ZALINGE-KH002	blood	M/S
Cam14	*sharpii*	Cambodia	Siem Reap, Angkor Centre for Conservation of Biodiversity	ACCB-A0058-1	oral swab	M/--
Ind01	*antigone*	India	Private collection: Lemgo/Germany	B1920	blood	M/--
Ind02	*antigone*	India	Private collection: Lemgo/Germany	B1921	blood	M/--
Ind03	*antigone*	India	Private collection: Lemgo/Germany	B2192	blood	M/S
Ind04	*antigone*	India	Private collection: Lemgo/Germany	B2822	blood	M/S
Ind05	*antigone*	India	Private collection: Lemgo/Germany	B2823	blood	M/S
Ind06	*antigone*	India	NMNH, Washington DC	USNM64453	toe pad	M/S
Ind07	*antigone*	India	Cincinnati Zoo	CinB299851	toe pad	M/--
Ind08	*antigone*	India	Cincinnati Zoo	CinB299851	toe pad	M/--
Mya01	*sharpii*	Myanmar	Minbya, Rakhine State	IPMB64768	blood	M/S
Mya02	*sharpii*	Myanmar	Maubin, Ayeyarwady Region	IPMB64769	blood	M/S
Mya03	*sharpii*	Myanmar	Maubin, Ayeyarwady Region	IPMB64770	blood	M/S
Mya04	*sharpii*	Myanmar	Maubin, Ayeyarwady Region	IPMB64771	blood	M/S
Mya05	*sharpii*	Myanmar	Einme, Ayeyarwady Region	IPMB64772	blood	M/S
Mya06	*sharpii*	Myanmar	Einme, Ayeyarwady Region	IPMB64773	blood	M/S
Mya07	*sharpii*	Myanmar	Nay Pyi Taw Zoo,	IPMB64774	blood	M/S
Mya08	*sharpii*	Myanmar	Nay Pyi Taw Zoo,	IPMB64775	blood	M/S
Mya09	*sharpii*	Myanmar	Yadanapon Zoo	IPMB64776	blood	M/S
Mya10	*sharpii*	Myanmar	Minbya, Rakaine State	IPMB64780	blood	M/S
Mya11	*sharpii*	Myanmar	Minbya, Rakaine State	IPMB64781	blood	M/S
Phi01	*luzonica*	Philippines	NMNH, Washington DC	USNM256982	toe pad	M/--
Tha01	*sharpii*	Thailand	Korat Zoo	275	blood	M/S
Tha02	*sharpii*	Thailand	Korat Zoo	280	blood	M/S
Tha03	*sharpii*	Thailand	Korat Zoo	282	blood	M/S
Tha04	*sharpii*	Thailand	Korat Zoo	283	blood	M/S
Tha05	*sharpii*	Thailand	Korat Zoo	288	blood	M/S
Tha06	*sharpii*	Thailand	Korat Zoo	292	blood	M/S
Tha07	*sharpii*	Thailand	Korat Zoo	294	blood	M/S
Tha08	*sharpii*	Thailand	Korat Zoo	295	blood	M/S

Only samples from captive birds in Germany and Australia (Lemgo Crane Collection and Cairns Tropical Zoo) and feathers from crane flocking sites in Australia and museum specimens in the United States were specifically collected for this project. All other samples assembled were derived from sets previously collected as part of other projects in Myanmar (captive zoo and monastery birds), Thailand (captive zoo birds), and Cambodia (wild-caught birds).

The blood sample collection protocol for captive German and Australian birds was for a three-person restraining team (all with significant experience in crane restraint and sampling) to catch the bird using a landing net; followed by immediate hooding and drawing ≤1mm of blood from the brachial vein (placing this immediately into 100% ethanol). In all cases restraint lasted less than 2 minutes. In Myanmar and Thailand, although sample collection was not part of this project, the collection protocol was consistent. In Cambodia, birds were wild caught using alpha chloralose, as part of a previous project and sampled as above, with an additional oral swab. One tissue (brain) sample from Cambodia was from a bird that had died recently from natural causes.

Shed feathers visibly free of soil and/or faecal contamination were gathered from crane flocking sites in Australia using tweezers and re-sealable plastic bags then refrigerated. Lightly-plucked chest feathers (3 to 5, ≤ 25mm) were obtained from restrained captive birds in Germany and Australia and refrigerated.

### Extraction

#### Nuclear DNA

DNA was extracted using the SDS/salting-out protocol of Miller *et al*. [34]. Dithiothreitol and Roti-PinkDNA (Carl Roth, Karlsruhe, Germany) were added in order to increase the yield. For the Cambodian blood and tissue samples, QIAGEN’s RNeasy Mini Kit was used, for an oral swab the QIAamp Viral RNA Kit. For the Thailand blood samples, DNA was extracted by using QIAGEN’s RNeasy Mini Kit. We amplified the ten microsatellite loci (Gamμ3, 18, 24,101b; GjM8, 13, 15, 48b; GR22, 25) used in our analysis of hybridization of the Brolga *Antigone rubicunda* and Australian Sarus Crane *A*. *antigone gillae* [19] which have been developed for other crane species [35,36], the Sarus Crane [32] and the Brolga [37], respectively. PCRs conducted in a volume of 10 μl contained 1 μl DNA (10–25 ng), 1 μl of 10 x NH_4_-based Reaction Buffer, 1.5–2.25 mM MgCl_2_ Solution (Table 2), 0.25 mM of each primer, 0.2 mM of dNTP, 0.04 μl of BioTaq DNA Polymerase (5 U/μl), 0.6 μl of 1% BSA and sterile ddH_2_O. If not successful with this first protocol, the MyTaq mix (all products from BIOLINE, London, UK) was used. The PCR profile started with an initial denaturation at 94°C, followed by 36 cycles of denaturation at 94°C, primer specific annealing (Table 2) and extension at 72°C each for 30 s, and a final elongation at 72°C for 30 min. Microsatellite alleles were separated on a 3130xl Genetic Analyzer using the GeneScan 600 LIZ Size Standard 2.0. Fragment sizes were determined manually in GeneMapper 4.0 (all three products from Applied Biosystems, Waltham, USA) as automatic calling with arbitrarily predefined bin width may give inconsistent results. In order to maximize accuracy of size determination, we repeated PCRs of samples with initially weak signals or which had rare variants. Eventually, PCR samples peculiar to different runs had to be loaded on the same plate to improve comparability.

#### Mitochondrial DNA

Where DNA quality allowed, we also sequenced large parts of copy 2 of the mitochondrial control region [38] using primers L16707 and H1247 [39] spanning a fragment of c. 1000 bp in *A*. *antigone* and 1150 bp in three specimens of *A*. *rubicunda*; one from the Gulf plains and two from the Atherton Tablelands (see [19]), which we used as outgroup in the phylogenetic analyses. In some specimens we had to target a shorter fragment using the forward primer L514 instead resulting in lengths of c.610 bp. PCRs were conducted using the MyTaq mix. The temperature profile for the long fragment comprised: 95°C for 3 min, 5 cycles of 95°C/15 s, 65°C/20 s and 72°C/25 s, 5 cycles 95°C/15 s, 60°C/20 s and 72°C/25 s, 30 cycles 95°C/15 s, 55°C/20 s and 72°C/25 s, and a final extension at 72°C for 5 min. For the short fragment the profile was similar and had 4, 4 and 32 cycles with respective annealing temperatures of 60°C, 55°C and 50°C. PCR products were cleaned using an exonuclease I/shrimp alkaline phosphatase mix. Cycle sequencing was then performed in 10 μl using the PCR primers and ABI’s Big Dye Terminator Ready Reaction Mix 3.1 of which 50% were replaced by halfBD (Merck). The thermal cycler profile followed the manufacturer’s suggestions except that the annealing temperature was lowered to 48° C. HighPrep DTR magnetic beads (Biozym) were used for purification of the sequencing reactions. The sequences were read on an ABI 3130xl Genetic Analyzer (Thermo Fisher Scientific Inc., Waltham, MA, USA). Raw sequences were edited in Geneious 10 (www.geneious.com) and BioEdit 7.0.5.3 [40], respectively, and aligned using the web version of MAFFT 7 [41].

### Statistical analysis

#### Nuclear DNA

FSTAT 2.9.3.2 [42] as well as GenePop 4.2 [43,44] were used to test the microsatellite data for Hardy-Weinberg equilibrium and to calculate gene diversity [45] and allelic richness [46] of subspecies. For population differentiation, the microsatellite data were analysed in two ways, (i) in a divisive approach without *a priori* designation of subspecies by Bayesian clustering using STRUCTURE 2.3.4 [47,48]; and (ii) by estimating differentiation of the nominal subspecies (except the single individual of *A*. *a*. *luzonica*), calculating pairwise F_ST_ values using FSTAT. STRUCTURE was run with K (number of clusters) ranging from one to six and ten replicates assuming the admixture model since the sequence analyses suggested that there had been admixture. We modelled both, uncorrelated and correlated allele frequencies as it was unclear which approach provided a better fit for the biological context. The Markov chains ran for 1 million generations after a burn in of 100,000. Structure Harvester 0.6.94 [49] was used to analyse the data following Evanno *et al*. [50]. Integrating the results of the replicated runs in STRUCTURE, the most likely assignment of individuals to clusters was inferred in CLUMPAK [51]. K-means clustering was applied to validate the Bayesian approach using GenoDive 2.0b23 [52] because it is free of population genetic assumptions in contrast to STRUCTURE. Individuals were clustered based on their allele frequencies according to the pseudo-F-statistic of Calinski and Harabasz [53] as described in Meirmans [54]. Finally, we estimated gene flow among subspecies based on F_ST_ and the private alleles approach of Barton and Slatkin [55] using GenePop (see [56,57]).

#### Mitochondrial DNA

Relationships among mitochondrial haplotypes were analysed using statistical parsimony/TCS [58] implemented in PopART [59] and MrBayes 3.2.6 [60], respectively. MrBayes was run using GTR+I+G identified as best fitting substitution model by jModeltest 2.1.4 [61] with default settings over 2 million generations with a 25% burnin. Effective sample sizes were > 700, potential scale reduction factors equalled 1.000 or 1.001, and the standard deviation of split frequencies was < 0.006 indicating convergence of parameter estimates and both parallel runs.

## Results

The nominate subspecies *A*. *a*. *antigone* had the highest diversity despite the lowest sample size, while *A*. *a*. *gillae* had comparatively lower diversity than the nominate subspecies (Table 3). *A*. *a*. *antigone* had four private alleles, two of them rare (only in one individual each and only heterozygous), *A*. *a*. *gillae* five, four of them rare (each in not more than 2 specimens and only heterozygous), and *A*. *a*. *sharpii* eight. Of these, five were rare (each in not more than three individuals and four only heterozygous). Three of the private alleles occurred only in Myanmar and another three in both Cambodia and Thailand. The single *A*. *a*. *luzonica* sampled had one allele that did not occur in any other subspecies. Deviations from the Hardy-Weinberg equilibrium at several loci in *A*. *a*. *antigone* and *A*. *a*. *sharpii* suggested that these subspecies are probably not panmictic, although we cannot rule out effects of genetic drift or selection. This was confirmed from the results of analysis using STRUCTURE and K-means clustering.

**Table 3:** PCR specifications and diversity of microsatellite loci. The subspecies are abbreviated by the first three letters (*ant*: *A*. *a*. *Antigone*; *gil*: *A*. *a*. *gillae*; *sha*: *A*, *a*. *sharpii*).

			N alleles			Gene diversity			Allelic richness		
Locus/dye	MgCl_2_ [mM]	T [°C]	*ant*	*gil*	*sha*	*ant*	*gil*	*sha*	*ant*	*gil*	*sha*
Gamμ3/FAM	1.5	59	5	2	5	0.839	0.115	0.768	4.741	1.570	4.364
Gamμ18/HEX	1.5	53	2	1	1	0.321	0.000	0.000	1.993	1.000	1.000
Gamμ24/HEX	2.25	60	7	5	5	0.567	0.227	0.534	4.000	2.290	3.291
Gamμ101b/HEX	2	59	8	2	7	0.821	0.393	0.799	5.399	1.985	5.048
GjM8/FAM	2	59	5	2	3	0.125	0.115	0.425	1.750	1.570	2.526
GjM13/HEX	2	60	5	2	4	0.875	0.488	0.591	4.849	1.999	3.209
GjM15/FAM	2	59	6	4	4	0.696	0.623	0.494	2.999	3.289	3.225
GjM48b/HEX	2	56	4	2	4	0.393	0.040	0.458	1.999	1.240	2.966
GR22/HEX	na	60	5	2	4	0.491	0.487	0.412	2.749	1.999	2.980
GR25/Cyanine3	na	60	3	3	2	0.339	0.486	0.496	1.993	2.237	1.999
		mean	5.0	2.5	3.9	0.547	0.297	0.498	3.247	1.918	3.062

**Fig 3:**
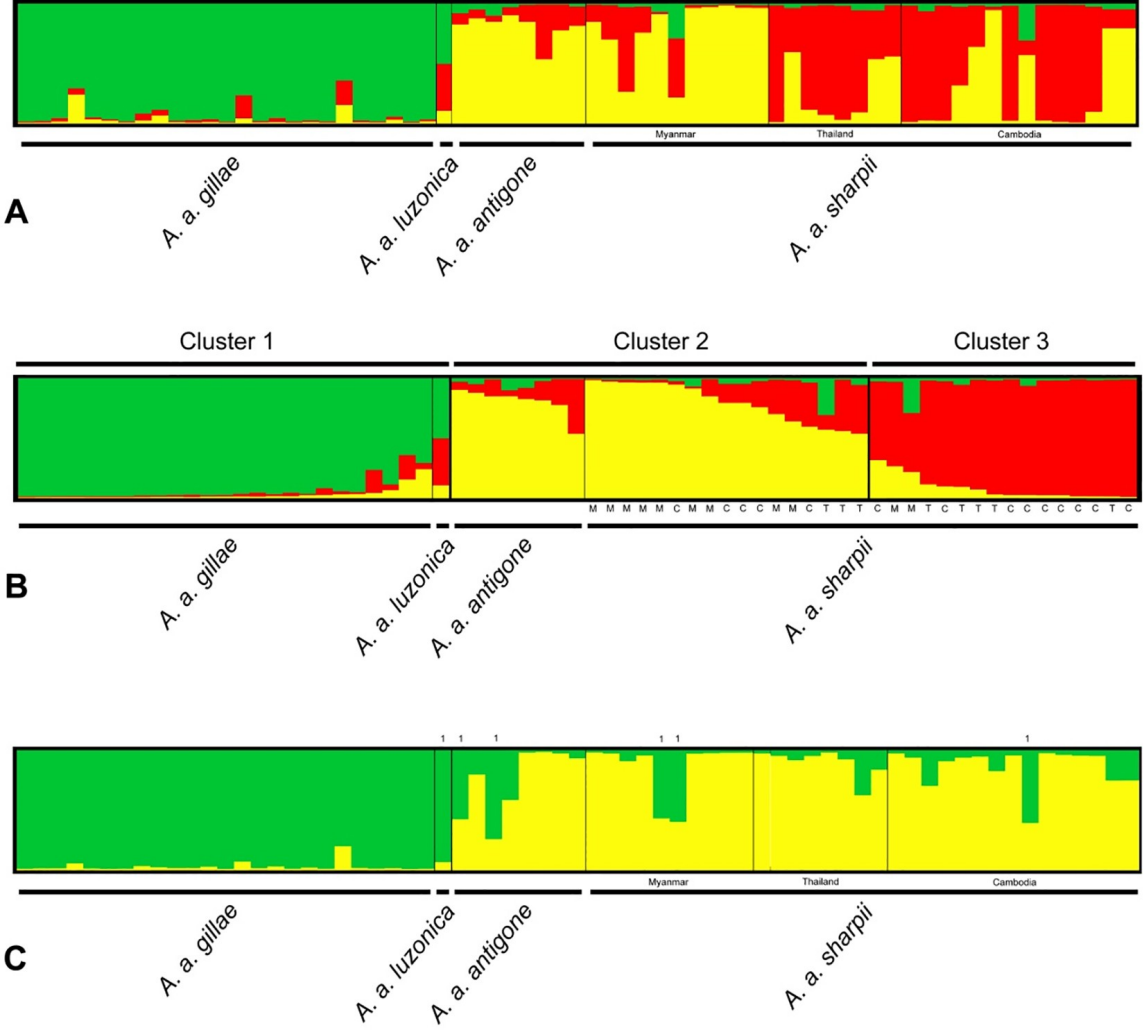
Bayesian cluster analyses of microsatellite data (STRUCTURE/CLUMPAK). A, B: assuming admixture and correlated allele frequencies, K = 3; y axis, membership coefficient from 0 (bottom) to 100 (top); in A, individuals are sorted by subspecies and country of origin; in B, samples are ordered by cluster, subspecies within clusters and membership coefficient Q (y-axis). For *A*. *a*. *sharpii*, the country of origin is indicated by the first letter (C = Cambodia, M = Myanmar, T = Thailand). C: assuming admixture and independent allele frequencies, K = 2; samples ordered by subspecies and country of origin within *A*. *a*. *sharpii*. Cluster 1 consists of all *A*. *a*. *gillae* as well as specimens labelled with 1.

According to Evanno *et al*.’s [50] ΔK criterion and assuming correlated allele frequencies, STRUCTURE identified three clusters, modelling independent allele frequencies only two (Fig 3; cluster composition as summarized by CLUMPAK Table 4). In both analyses, all *A*. *a*. *gillae* fell into one cluster together with the *A*. *a*. *luzonica* specimen. Assuming independent allele frequencies, the cluster with these subspecies also contained three specimens of *A*. *a*. *sharpii* and two Indian individuals. For both models, a solution with four clusters had the highest likelihood but the composition of the clusters was less meaningful, apart from grouping all Australian individuals together. K-means clustering also divided the sample set into two clusters (Table 4), one consisting of 23 *A*. *a*. *gillae*, one *A*. *a*. *sharpii*, and the single *A*. *a*. *luzonica*, and the other of two *A*. *a*. *gillae*, all *A*. *a*. *antigone* from India, and the remaining *A*. *a*. *sharpii*. Both Bayesian clustering (assuming admixture and independent allele frequencies) as well as k-means clustering converged to very similar solutions. The STRUCTURE bar plots also reflect the higher genetic diversity in the Asian subspecies as summarized by the standard population genetic parameters above and in Table 2.

**Table 4:** Cluster analyses (codes from Table 2 above).

Bayesian clustering assuming population admixture					k-means clustering	
correlated allele frequencies			independent allele frequencies			
Aus01	Cam05	Cam01	Aus01	Cam01	Aus01	Aus08
Aus02	Cam06	Cam02	Aus02	Cam02	Aus02	Aus20
Aus03	Cam08	Cam03	Aus03	Cam03	Aus03	Cam01
Aus04	Cam13	Cam04	Aus04	Cam04	Aus04	Cam02
Aus05	Cam14	Cam07	Aus05	Cam05	Aus05	Cam03
Aus06	Ind01	Cam09	Aus06	Cam06	Aus06	Cam04
Aus07	Ind02	Cam10	Aus07	Cam07	Aus07	Cam05
Aus08	Ind03	Cam11	Aus08	Cam09	Aus09	Cam06
Aus09	Ind04	Cam12	Aus09	Cam10	Aus10	Cam07
Aus10	Ind05	Mya03	Aus10	Cam11	Aus11	Cam09
Aus11	Ind06	Mya06	Aus11	Cam12	Aus12	Cam10
Aus12	Ind07	Tha01	Aus12	Cam13	Aus13	Cam11
Aus13	Ind08	Tha03	Aus13	Cam14	Aus14	Cam12
Aus14	Mya01	Tha04	Aus14	Ind02	Aus15	Cam13
Aus15	Mya02	Tha05	Aus15	Ind04	Aus16	Cam14
Aus16	Mya04	Tha06	Aus16	Ind05	Aus17	Ind01
Aus17	Mya05		Aus17	Ind06	Aus18	Ind02
Aus18	Mya07		Aus18	Ind07	Aus19	Ind03
Aus19	Mya08		Aus19	Ind08	Aus21	Ind04
Aus20	Mya09		Aus20	Mya01	Aus22	Ind05
Aus21	Mya10		Aus21	Mya02	Aus23	Ind06
Aus22	Mya11		Aus22	Mya03	Aus24	Ind07
Aus23	Tha02		Aus23	Mya04	Aus25	Ind08
Aus24	Tha07		Aus24	Mya07	Cam08	Mya01
Aus25	Tha08		Aus25	Mya08	Phi01	Mya02
Phi01			Cam08	Mya09		Mya03
			Ind01	Mya10		Mya04
			Ind03	Mya11		Mya05
			Mya05	Tha01		Mya06
			Mya06	Tha02		Mya07
			Phi01	Tha03		Mya08
				Tha04		Mya09
				Tha05		Mya10
				Tha06		Mya11
				Tha07		Tha01
				Tha08		Tha02
						Tha03
						Tha04
						Tha05
						Tha06
						Tha07
						Tha08

Differentiation among subspecies based on F_ST_ estimates revealed that *A*. *a*. *antigone* and *A*. *a*. *sharpii* were considerably closer to each other (F_ST_ = 0.086) than either were to *A*. *a*. *gillae* (F_ST_ = 0.282 and 0.168, respectively). These F_ST_ values translated into gene flow estimates of 2.66 migrants per generation between *A*. *a*. *antigone* and *A*. *a*. *sharpii*, 0.64 between the nominate subspecies and *A*. *a*. *gillae*, and 1.24 between *A*. *a*. *sharpii* and *A*. *a*. *gillae*. The private alleles approach estimated 0.71, 0.44, and 0.53 migrants, respectively. This again emphasises the somewhat isolated position of the Australian subspecies.

**Fig 4:**
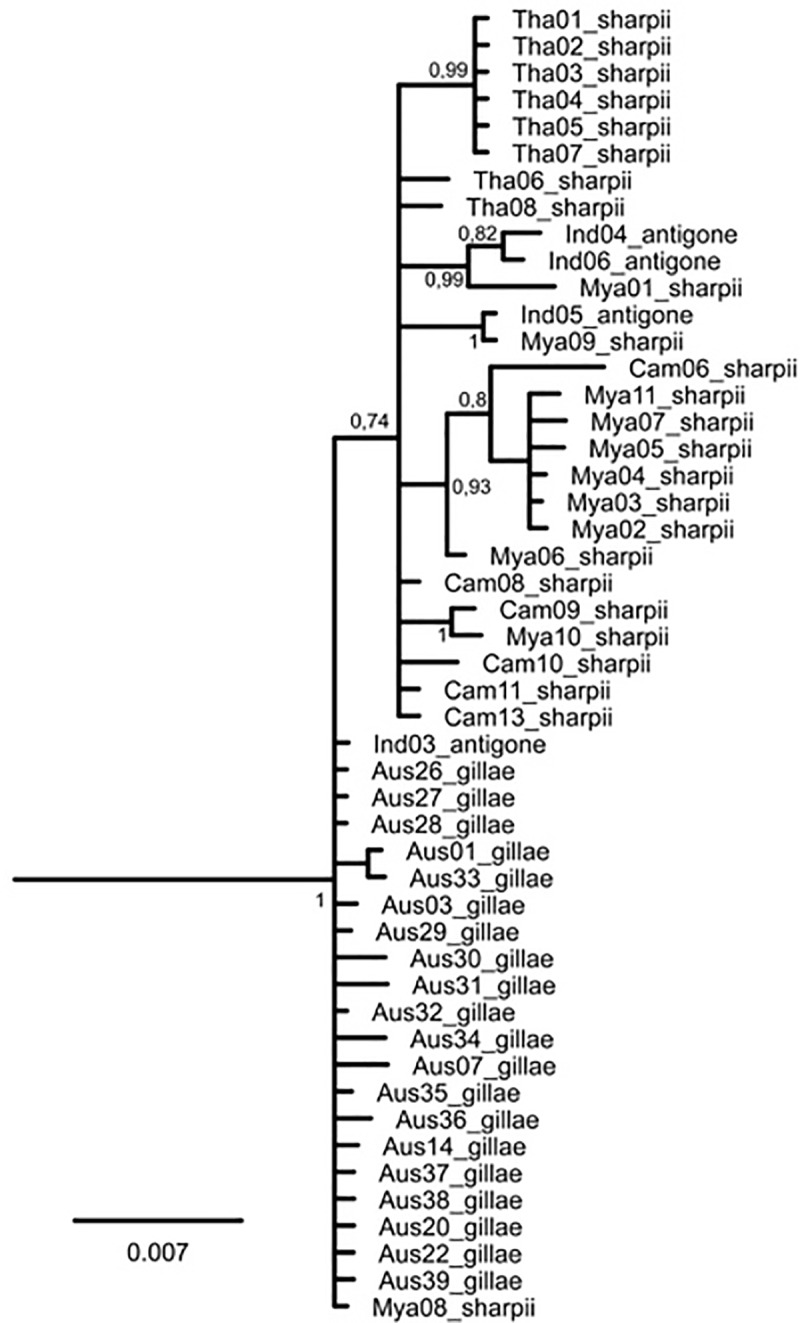
Phylogenetic tree for three subspecies of the Sarus Crane *A*. *antigone*; based on mtDNA sequences resulting from Bayesian analysis. Posterior probabilities are given if > 0.70 (for sample codes see Table 2; outgroup (*A*. *rubicunda*) pruned off.).

**Fig 5:**
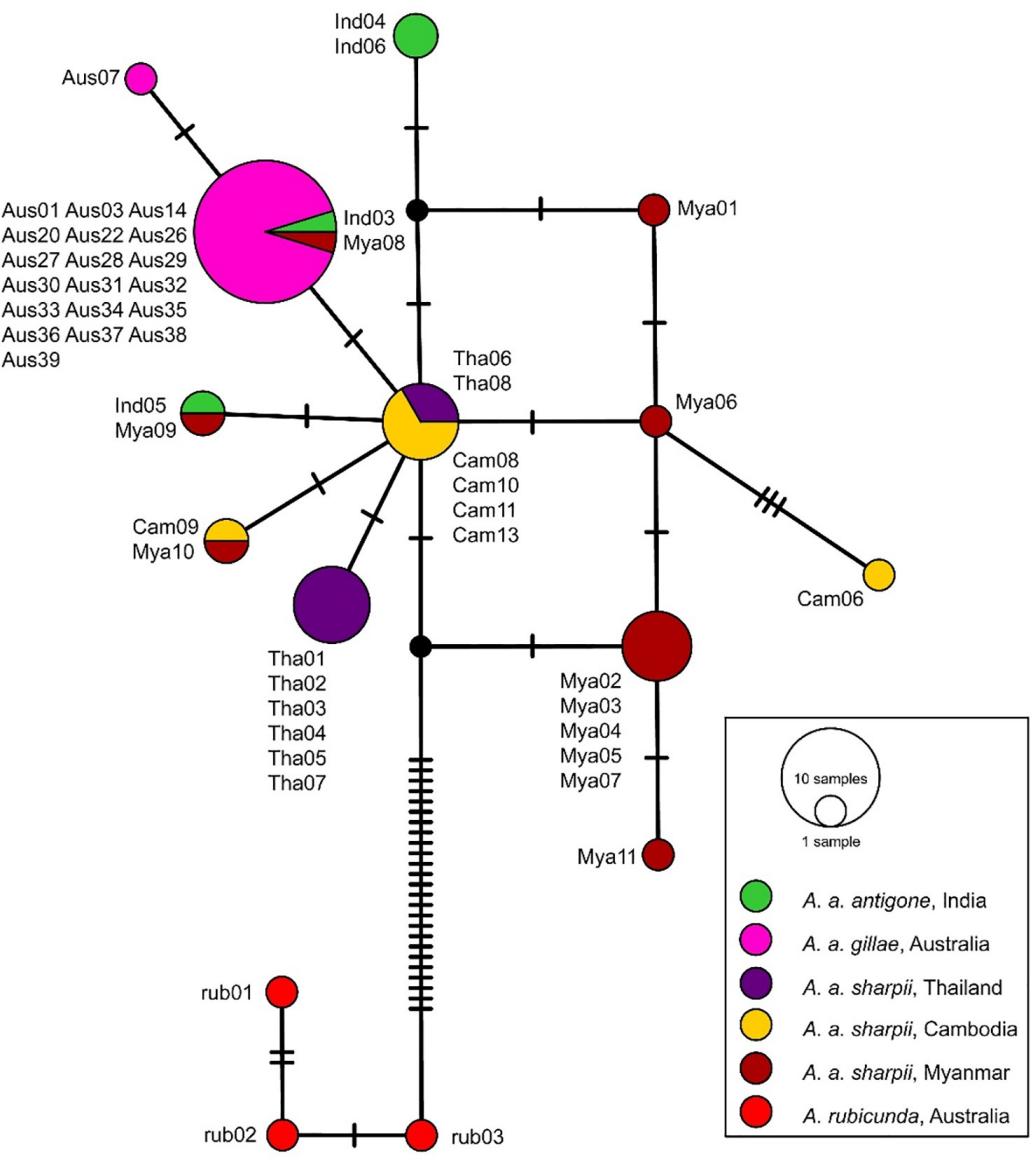
TCS network for five populations of the Sarus Crane *A*. *antigone* based on mtDNA sequences compared to the Brolga *A*. *rubicunda* (for sample codes see Table 2).

The alignment of the control region 2 sequences comprised 1155 positions. A major difference between *A*. *rubicunda* and *A*. *antigone* were two indels comprising 46 and 95 positions, respectively, which were present in the former and absent in the latter species. Apart from these, *A*. *rubicunda* differed by at least 28 mutations from *A*. *antigone*, rendering the latter monophyletic in the Bayesian tree reconstruction (Fig 4), which is also illustrated by the TCS network (Fig 5). However, both tree and network agreed that no subspecies of *A*. *antigone* was monophyletic. Both reconstructions suggested an ancestral polymorphism and/or repeated introgression, meaning that there had been at least limited gene flow among the subspecies. Given the overall low differentiation across *A*. *antigone*, resulting in low posterior probabilities (i.e. node support) and the low sample size of the nominate subspecies, inferring evolutionary directions is not possible.

## Discussion

Our analyses differ from earlier work [31,32] by having a larger sample size and in sequencing a highly variable part of the mitochondrial control region [39] instead of protein coding genes [31], thereby providing better phylogenetic resolution. Similarly to [31], we found that Sarus Crane subspecies and populations were not monophyletic (probably due to an ancestral polymorphism and/or introgression) and microsatellite variation in *A*. *a*. *antigone* and *A*. *a*. *sharpii* overlapped significantly [32]. However, we have established that *A*. *a*. *gillae* is far more distinct from *A*. *a*. *antigone* and *A*. *a*. *sharpii* than previously thought, irrespective of the clustering method and the model assumptions used in Bayesian clustering. This was also confirmed by F-statistics and gene flow estimates. We have also shown that the single *A*. *a*. *luzonica* specimen we have hitherto been able to sample was more similar to *A*. *a*. *gillae* than the geographically closer *A*. *a*. *sharpii*. The first finding has potential implications for definitions of subspecies, the second in relation to better understanding the phylogeography of the species and potential sourcing of birds for any Philippine reintroduction. We are well aware how problematic any conclusions based on a single specimen might be but given that Philippine Sarus Cranes are extinct and the scarcity of museum material, no alternative approach is available.

Given there are now attempts to define subspecies under law [62], there is a need for far greater understanding of just how much weight should be given to genetic data, particularly where genetic variation appears to be lacking. While patterns of crane morphological variation have not been reflected in marked genetic differences between populations [63,64,65], the findings of Jones *et al*. [32] could have been used to suggest that the variation in Sarus Cranes is clinal without distinct breaks in genetic variability. Our results suggest that simply by looking at a slightly different part of the genome with a larger sample size a different conclusion would have been drawn. This is relevant for current Australian policy, which has not been consistent. For example, Schodde and Mason [66] diagnosed new subspecies of Southern Emu-wren *Stipiturus malachurus* and Eastern Bristlebird *Dasyornis brachypterus* on the basis of morphological discontinuities. Despite genetic differences in the emu-wren failing to match morphology [67], threatened subspecies continue to be recognised under legislation [68]. A similar level of variation in the Eastern Bristlebird [69] has meant that there has been no recognition of the northern form of Eastern Bristlebird *D*. *b*. *monoides* [66], of which 40 individuals are thought to survive [70] but for which conservation effort has been inconsistent [71]. Were rigid definitions of subspecies enforced by law, as now being argued in the USA [62], with the level of knowledge previously available from Jones *et al*. [32], Sarus Crane subspecies might not have been eligible for conservation as separate subspecies. Our results confirm the position of Patten and Remsen [7] that synonymising subspecies can be highly problematic without testing hypotheses using multiple data sources, as advocated by integrative taxonomy [72]. We therefore wish to stress that our findings are not a final verdict on phylogeographic differentiation in the Sarus Crane, which requires further morphological and genetic work to complement our analyses.

Although first formally noted in Australia in the 1960s [29], Sarus Cranes have been in the country long enough to have been given a Wik (Cape York Aboriginal language group) name, meaning ‘the Brolga that dipped its head in blood’ [73]. Wood and Krajewski [31] have suggested that Sarus Cranes first arrived in Australia 37,500 years ago, when sea levels were 40 m lower than today [74], permitting development of a savanna corridor that extended both north and south of the equator across the Sunda plain [75], much of which would have been lost by rising sea levels at the start of the Holocene (about 10,000 years ago). In an Australian context, this corridor [76] would have ended not far north of the Pleistocene Lake Carpentaria, around which it is thought there would have been savannas structurally similar to those used by Sarus Cranes in northern Australia. It is also true that lower sea levels would have narrowed the distance between the Philippines, Borneo/Peninsula Malaya (via Palawan) and Indochina [77] and hence potentially also brought *A*. *a luzonica* and *A*. *a*. *sharpii* into closer proximity. However, especially given the shifting of the courses of the Mekong [78], it is likely that facing coastal regions of both Indochina and the Philippines would have been mainly comprised of closed forest, making sub-specific contact more difficult.

## Conclusions and recommendations

We have shown that *A*. *a*. *gillae* differs significantly from the *A*. *a*. *antigone* and *A*. *a*. *sharpii* genetic cline described by others. Where once *A*. *a gillae* might have been considered part of this cline, more detailed analysis has revealed greater structure. This has relevance to the wider debate about subspecies, suggesting that the level of genetic analysis required before subspecies are dismissed needs to be carefully considered, and wherever feasible triangulated with information gleaned from other character traits.

That the single sample from *A*. *a luzonica* clustered with *A*. *a*. *gillae* hints at the potential for a close evolutionary relationship. Should reintroduction of Sarus Cranes to the Philippines be deemed desirable and viable, subject to further research on the genetic affinities of *A*. *a*. *luzonica*, Australia might be an appropriate source of birds.

Whilst Hachisuka [27] found that Philippine birds were significantly smaller than those on the south-Asian mainland, the general case for insular dwarfism is equivocal [79]. As we had access to only one individual of *A*. *a*. *luzonica*, further genetic work on samples from Philippine museum specimens could help to clarify the status of this subspecies and its potential to shed further light on the phylogeography of Sarus Cranes.
